# The Integration of Recipes with a Standardizable Food Description FACET for Cadmium Exposure Risk Assessment

**DOI:** 10.3390/ijerph16234825

**Published:** 2019-11-30

**Authors:** She-Yu Chiu, Hsin-Tang Lin, Min-Hua Lin, Wen-Chao Ho, Pau-Chung Chen, Hui-Ying Huang

**Affiliations:** 1 Department of Nutrition, China Medical University, Taichung 40402, Taiwan; koca345678@gmail.com (S.-Y.C); minhua5356@gmail.com (M.-H.L.); emily572005@gmail.com (H.-Y.H.); 2 Graduate Institute of Food Safety, National Chung Hsing University, Taichung 40200, Taiwan; 3 Department of Public Health, China Medical University, Taichung 40402, Taiwan; 4 Department of Nursing, Asia University, Taichung 41354, Taiwan; 5 Department of Public Health, National Taiwan University, Taipei 10617, Taiwan; pchen@ntu.edu.tw

**Keywords:** cadmium, consumption database, food recipes, risk assessment

## Abstract

Existing food classification and description systems provide users with limited information related to exposure assessment. Our aim in this work is to propose a standardized food description facet called the Taiwan Food Recipe (TFR) system as an emerging tool for food composition, with detailed food ingredient information, including names, proportions, weights of uncooked and cooked foods, etc. The composite foods listed in the Taiwan Nutrition and Health Survey were collected into a list and as consumption data. The TFR system is intended to help analysts reduce potential estimation bias, where, for example, risk assessment results may be overestimated or underestimated due to the complexity of the composition in the composite foods. Based on a Taiwanese food database, we further illustrate and demonstrate how the TFR system can be applied to the assessment of risk of cadmium (Cd) exposure in rice ingredients in the composite food products. In the original system (HFDFC system), the composite food intakes used total weight to estimate the hazard index (*HI*) of cadmium in the exposure risk assessment, but the percentage of rice was not 100%. The proposed TFR system estimates the percentage of rice and actual intakes in composite foods. Fried rice, sushi, and rice balls in the study were the most common foods containing rice and had higher consumption rates among Taiwan’s rice-based composite foods. The *HIs* of fried rice, sushi, and rice balls were 0.09, 0.10, and 0.13, respectively, in the HFDFC system. In the TFR system, the *HIs* of rice in fried rice, sushi, and rice balls were 0.06, 0.04 and 0.05, respectively. The *HI* of other components in fried rice, sushi, and rice balls were 0.03, 0.06 and 0.08, respectively. More precise *HIs* were thus shown. The TFR system contributes to global food classification and description systems by providing an appropriate, standardized, and generalized framework for exposure assessments.

## 1. Introduction

Contemporary food classification and description databases provide users with comprehensive information, yet it is difficult for risk assessment analysts to efficiently estimate food intake. For example, Foodex2 is a food classification and description database developed by the European Food Safety Authority. It comprises standardized identification and characterization of food and feed items in order for users to retrieve detailed food information [1,2,3]. In Foodex2, the rice compound products in Austria in 2005 were classified into composite dishes (level 1), pasta and rice dishes (level 2), rice-based dishes (level 3), and rice pudding dishes (level 4) [4]. Because the database only provides overall consumption data for rice pudding, analysts who need detailed food component information for rice pudding for the purpose of risk assessment may not be able to reach a proper conclusion because of insufficient food data. This situation is also common in the Asia–Pacific region, where composite foods are the major part of the catering culture. For instance, the Harmonized, Food Description Incorporated, Food Classification system (hereafter, the HFDFC system) is a popular food classification and description database in Taiwan. Because the HFDFC system only classifies food intake by food ingredients [5], it is quite time consuming for risk assessment analysts to estimate food intake from a specific food. In addition, a biased conclusion from the risk assessment could be reached because Chinese cuisine varies substantially with the recipe. For example, the degree of heavy metal substances in a sushi product can vary with cooking methods such as pickling, simmering, and raw consumption. In this case, analysts relying on the HFDFC system might overestimate or underestimate the degree of heavy metal substances in a sushi product. We complement the existing databases by establishing a standardized food description FACET based on recipes and demonstrate how useful it is in terms of risk assessment.

The FACET is aimed toward the construction of a standardized recipe-based food description FACET called the Taiwan Food Recipe system (hereafter, TFR), which provides ingredient information including names, proportions, weights of uncooked and cooked foods, etc. This standardized food description FACET can be integrated into the HFDFC system and provides users with useful information for the purpose of food risk assessment. We further illustrate the usefulness of the TFR system in risk assessments of cadmium, a heavy metal that is harmful to the human body, in the rice in the composite food. Rice is one of the most popular food crops in Taiwan as well as in the Asia–Pacific region [6]. It may contain polluted substances accrued during the metabolic process if it is irrigated with polluted water and sediments. Related studies use the degree of cadmium concentration in rice as an important indicator in the evaluation of ecological and health risks [7,8,9,10]. In this study, we demonstrate how users can apply the TFR to the cadmium estimation in the rice ingredients of composite food.

### 1.1. A Comparison of Food Classification Systems with the TFR System

#### 1.1.1. Food Classification Systems

In Europe, the European Food Information Resource has been developing a food recipe system since 2005. It includes food descriptors, portion sizes, standard recipes, and yield factors [11]. In 2011, the European Food Safety Authority (hereafter, the EFSA) established the Foodex2 database for exposure assessment. Foodex2 contains useful food information including food ingredients and weight, etc. A good function of this database is that it connects food information with food descriptions (major ingredients) in terms of standardized codes, which is useful to retrieve standardized composite food information when users are conducting an exposure assessment [1]. To facilitate the use of food information, the EFSA improved the Foodex2 database in 2015 by expanding the terminology, revising the definitions and FACET, and creating a specific reporting hierarchy and part-nature-based generic terms [2].

In the U.S., the National Center for Health Statistics (hereafter, the NCHS) was authorized under the National Health Survey Act of 1956 to collect national-level statistics on a wide range of health issues (See https://www.ncbi.nlm.nih.gov/books/NBK217716/#ddd00047). The NCHS has conducted a series of National Health and Nutrition Examination Surveys (hereafter, NHANES). The NHANES I conducted between 1971 and 1974 first covered the topic of the nutritional status of foods. Recipes were included in the NHANES II questionnaires administered between 1976 and 1980. The data from the NHANES II helped users calculate the macronutrient, fat, vitamin, and mineral content of the foods reported as consumed [12]. Thereafter, the U.S. Environmental Protection Agency (hereafter, the EPA) further developed a food database called the Food Commodity Intake Database (hereafter, the FCID) to improve the utility of food consumption surveys for the purpose of pesticide dietary exposure assessments [13]. The FCID translates food consumption into consumption of U.S. EPA-defined food commodities.

In Taiwan in 2012, the Taiwan Food and Drug Administration (hereafter, the TFDA) authorized the National Health Research Institutes (hereafter, the NHRI), Academia Sinica, and the China Medical University to develop and maintain the Taiwan National Food Consumption Database (hereafter, the TNFCD) [14]. In addition to providing food intake information, the TNFCD classifies food ingredients into four layers based on dietary habits in Taiwan. The first layer (Level-One) includes seventeen categories of food products (e.g., composite foods; vegetables, etc.). The second layer (Level-Two) includes the sub-categories of the Level-One categories (e.g., rice composite foods, wheat composite foods, other composite foods, leafy vegetables, rhizomes, cruciferous vegetables, etc.). The third layer (Level-Three) includes the items in the Level-Two sub-categories (e.g., rice balls, fried rice, dumplings, lettuce, radishes, broccoli, etc.). The fourth layer (Level-Four) includes sub-items of the Level-Three items (e.g., butter crunch lettuce, leaf lettuce, Boston lettuce). In 2016, the TNFCD incorporated a food description system with diversified food information. In 2017, the TFDA added food descriptions to the TNFCD and renamed it as the HFDFC, an extensive system used for exposure assessment [5]. The HFDFC contains 17 categories in Level one, 67 sub-categories in Level-Two, 199 items in Level-Three, 131 sub-items in Level-Four, and six FACETs in the description system for exposure assessment [5]. All levels are coded with the symbol “#” to establish a link between the food classification items and the food description items. The links between the two food description items were established with the symbol “$” in the HFDFC system. For instance, A010101#F01.1 means white rice (A010101) in the food classification system and is the plant origin of the food source FACET (F01.1). Food intakes related to composite foods are only included in Level-One, Level-Two, and Level-Three.

#### 1.1.2. The Taiwan Food Recipe System (TFR)

Recipes are a key element in food culture [15,16] because they contain important information about food ingredients and preparation methods [17]. The role of recipes is especially important in Asia because the cooking methods for Asian-style foods are relatively complicated as compared to Western-style foods. However, the existing food classification systems fail to incorporate recipes, which makes it more difficult for analysts to accurately assess potential exposure to hazardous substances in the food products.

**Figure 1:**
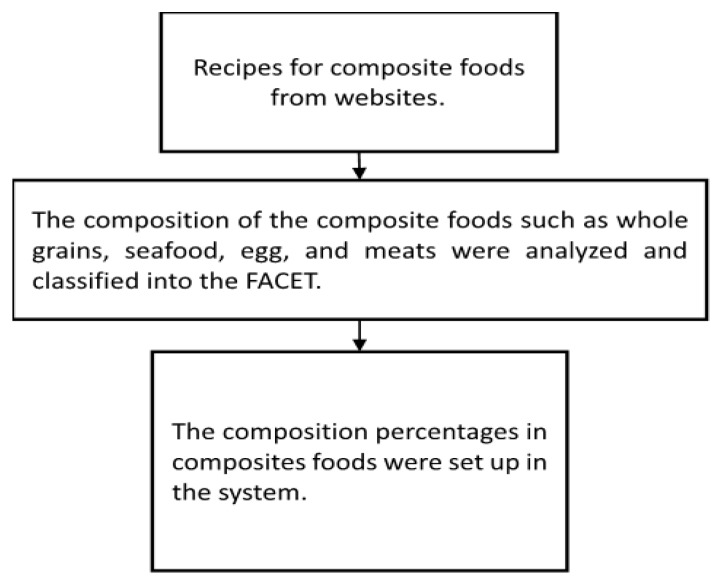
Schematic diagram of Taiwan Food Recipe (TFR) system.

We proposed a supplementary food description FACET that integrates recipes into the HFDFC system, called the Taiwan Food Recipe (TFR) system (Figure 1). The TFR system comprises recipes of composite food from websites. The composition of the composite foods such as whole grains, seafood, egg, and meats were analyzed and classified into the FACET. The composition percentages in composites foods were then set up in the system. We retrieved food intake data from a dietary database called the Taiwan Nutrition and Health Survey (hereafter, the NAHSIT) [18], which contains food consumption data for Taiwanese people of all ages between 2005 and 2012. The HFDFC system calculates food intake from the NAHSIT food consumption data so that the TFR system can be integrated into the HFDFC system. The recipes for the TFR system were retrieved from three popular, reliable websites: (1) *icook*; (2) *ytower*; (3) *inyoung99*, *icook,* and *ytower* are popular, as evidenced by their high click-through rates [19,20]. *inyoung99* was co-developed by the Taiwan Ministry of Health and Welfare, National Taiwan University, and the Taiwan Geographic Information Systems Center [21].

The TFR system was built on the composite food categories in the HFDFC system. In the HFDFC system, the Level-Two sub-categories of composite foods include (1) rice composite foods [HFDFC code: *O01*], (2) wheat composite foods [HFDFC code: *O02*], and (3) other composite foods [HFDFC code: *O03*]. To synchronize the TFR system with the HFDFC system and Chiu et al. (2018) (Chiu et al., 2018 encoded six food descriptions (F01–F06) to synchronize their system with the HFDFC system [5]), we created a TFR identification code, called “*F07*,” representing the food composition mainly designed for assessment of food risks. We used the symbol “#” to connect “*F07*” to the corresponding coded Level-Two categories in the HFDFC system.

The food ingredients suggested by the recipes for cooking the composite foods were then grouped into fourteen coded food descriptors, which include whole grains [TFR sub-code: 01], water [TFR sub-code: 02], beans [TFR sub-code: 03], seafood [TFR sub-code: 04], eggs [TFR sub-code: 05], meats [TFR sub-code: 06], milk [TFR sub-code: 07], vegetables [TFR sub-code: 08], fruits [TFR sub-code: 09], nuts [TFR sub-code: 10], fats [TFR sub-code: 11], beverages [TFR sub-code: 12], wines [TFR sub-code: 13], and seasonings [TFR sub-code: 14]. We used the symbol “*$*” to establish a connection between two coded food descriptors.

For each composite food entry, we performed a Monte Carlo method adjusted for 10,000 iterations to estimate the raw weight, the cooked weight, and the percentages of these against the average weight of each food ingredient in all of the recipes under consideration (The Monte Carlo method is an extensive calculation algorithm that relies on repeated random sampling to obtain the distribution of numerical results [22]. It can be used to simulate the estimation of point-to-area estimates in a random manner). As a result, the TFR system contained both the cooked and uncooked weight information for the main ingredients in the recipes for the three Level-two categories of composite foods in the HFDFC system.

We take pizza as an example to illustrate how the TFR system connects to the HFDFC system. In the HFDFC system, pizza is coded *O0206* at the Level-Three layer in the HFDFC system. The main ingredients in pizza are flour, seafood, meats, and vegetables. These ingredients are classified into whole grains [TFR sub-code: 01], seafood [TFR sub-code: 04], meats [TFR sub-code: 06], and vegetables [TFR sub-code: 08] in the HFDFC system. Users who are looking for the food intake from a pizza can look up the combined code: *O0206# F07.1$ F07.4$ F07.6$ F07.8*.

## 2. Methods

### 2.1. Applying the TFR System to a Cadmium Risk Assessment for Rice

#### Procedures

We further demonstrated the usefulness of the TFR system in risk assessment by conducting a cadmium risk assessment for fried rice products, sushi products, and rice ball products. Following the World Health Organization (WHO) protocols, we performed a system risk assessment, also called a non-cancer risk assessment, to examine the likelihood of excessive cadmium in the three food products under consideration [23].

We obtained dietary consumption data from NAHSIT for the period of 2005–2012. We manually collected a list of food products sold in the market from 2005–2012. Data for adults with average body weight were retrieved from the HFDFC. We focused on Taiwanese citizens between ages of 19 and 65. Data for cadmium concentrations were retrieved from Syu et al. [24]. Data for oral exposure to cadmium were retrieved from the United States Environmental Protection Agency (hereafter, the EPA) (reference number: CASRN 7440-43-9).

**Figure 2:**
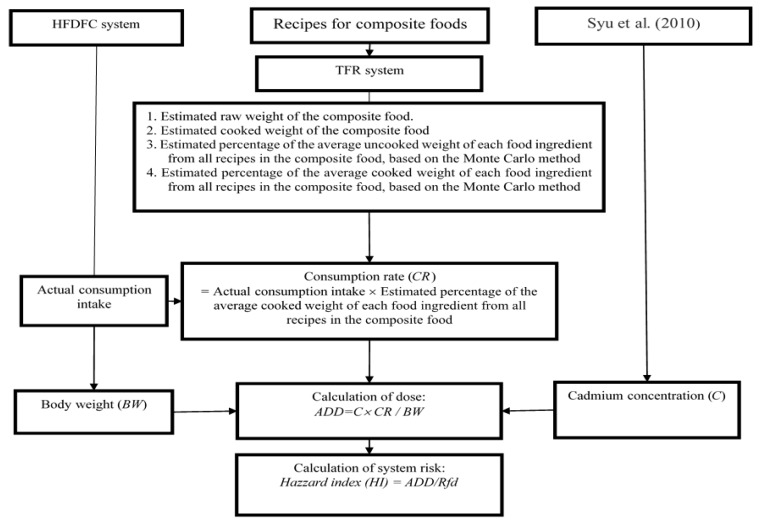
The application of the TFR system application on a cadmium risk assessment for rice.

Figure 2 depicts the research framework. First, we estimated the experimental daily consumption rate (*CR*) per person for the food ingredients in the composite foods by using the combined codes to access the estimated percentage of the average cooked weight of each food ingredient from all recipes in the composite food from the TFR system and the actual consumption intake from the HFDFC system. For each of these three composite food entries, we then used Equation (1) to estimate the average daily dose (*ADD*) per person allowed for tolerable cadmium exposure.

(1) $$ADD=\frac{C\times CR}{BW}$$

where *C* refers to the cadmium concentration in the food ingredients retrieved from Syu et al. [24], measured in mg/kg. *CR* refers to the daily consumption rate of a food ingredient per person. *BW* refers to the average body weight in kilograms among adults between the ages of 19 and 65.

Expressed using Equation (2), we then used the *ADD* estimated from Equation (1) as well as the tolerable oral exposure to cadmium (*RfD*) to estimate the hazard index (*HI*) for the food ingredients of the composite food entry. An *HI* value that exceeds one represents a high level of exposure to hazardous substances that can damage human organs [25].

(2) $$HI=\frac{ADD}{RfD}$$

where *RfD* refers to a tolerable level of oral exposure to cadmium, as retrieved from the EPA.

## 3. Results

TFR code played an important role in the standardization of the complex composite food shown in Table 1 and the link between the composition of the composite foods and the FACET using the TFR coding system. The formulation of the TFR codes was done for fried rice products, sushi products, and rice ball products. Fried rice products, sushi products, and rice ball products are coded with *O010301*, *O010302*, and *O010303* in the HFDFC system, respectively. There were six fried rice products, 31 sushi products, and 79 rice ball products in high demand in the market during the period from 2005–2012. For each of these food products, we identified the main food ingredients from the recipes. We then classified the main ingredients according to the TFR sub-description coding system. To make fried rice products, for instance, the recipes mainly require rice, anchovy, larvae, salmon, egg, pork, beef, sweet pepper; cabbage, and salt soy sauce. These food ingredients were classified into five sub-descriptions in the TFR coding system, including whole grains [*F07.1*], seafood [*F07.4*], egg [*F07.5*], meat [*F07.6*], vegetable [*F07.8*], vegetable (*F07.8*), and seasoning (*F07.14*). The combined code for fried rice products in the TFR coding system was therefore “*O010301# F07.1$ F07.4$ F07.5$ F07.6$ F07.8$ F07.14*”. Likewise, the combined codes for sushi products and rice ball products were “*O010302#F07.1$F07.4$F07.6$F07.8$F07.9$F07.14*” and “*O010303#F07.1$ F07.4$ F07.6$ F07.8$ F07.14*”, respectively.

In order to conduct a Cd risk assessment of the rice in rice-based composite foods, we then used these combined codes to retrieve the estimated percentage of the average cooked weight of each food ingredient from all recipes in the fried rice products, the sushi products, and the rice ball products, respectively. The adjusted weights of the rice and other TFR sub-descriptions for the fried rice, sushi, and rice ball products are listed in Table 2. The mean adjusted rice weight for the fried rice products was 176.53 g, which accounted for 65.6% of the total weight of the fried rice products. The mean adjusted rice weight for the sushi products was 77.23 g, which accounted for 43.5% of the total weight of the sushi products. The mean adjusted rice weight for the rice ball products was 461.07 g, which accounted for 36.9% of the total weight of the rice ball products. The food intakes for different rice composite foods could be converted using the ratio of rice in the table to obtain more accurate results.

The consumption rates (*CR*) and the cadmium hazard indexes (*HIs*) of the rice composite foods in HFDFC system and TFR system are shown in Table 3. Table 3 also provides (*CR*) and (*HIs*) by gender in HFDFC system and TFR system. Panel A of Table 3 shows the estimation process for the consumption rates. Column (1) of Panel A contains the *CR* estimated from the HFDFC system. In column (1), the mean values of *CR* for the fried rice, sushi, and rice ball products were 127.5 (g/day), 177.87 (g/day), and 188.06 (mg/kg/day), respectively. On average, men consumed more fried rice and sushi but fewer rice balls than women during the study period. Column (4) reports the CR for the rice ingredient of the composite products. In column (4), the mean values of *CR* for the rice ingredients in the fried rice, sushi, and rice ball products were 83.64 (g/day), 73.77 (g/day), and 69.39 (g/day), respectively.

The consumption rates were then used to determine the cadmium hazard indexes of the composite products. Traditionally, analysts can only estimate the cadmium hazard indexes for composite products using the HFDFC system. Using the TFR system, analysts can estimate the cadmium hazard indexes for the rice ingredient in the composite product. Panel B of Table 3 reports the cadmium hazard indexes using the HFDFC system and those using the TFR system.

In column (1) of Panel B, the mean values of the *HI* for the fried rice, sushi, and rice ball products were 0.09, 0.10, and 0.13, respectively. All *HI* values for these three composite products were less than one, representing low potential exposure to cadmium. Columns (2) and (3) of Panel B report the *HI* values for the rice ingredient and other food ingredients in the composite products. In column (2) of Panel B, the mean *HI* values for the rice ingredients of the fried rice, sushi, and rice ball products were 0.06, 0.04, and 0.05, respectively. In column (3) of Panel B, the mean *HI* values for the rice ingredients in the fried rice, sushi, and rice ball products were 0.03, 0.06, and 0.08, respectively. For the fried rice and rice ball products, the potential exposure to cadmium for the rice ingredients was higher than that for other ingredients. By contrast, the potential exposure to cadmium for the rice ingredients in the sushi product was lower than that for the other ingredients. On average, men who prefer fried rice and sushi products encountered higher exposure to cadmium from the rice ingredients than women. By contrast, women who prefer rice ball products encountered higher exposure to cadmium for the rice ingredients than men. Overall, Table 3 demonstrates how analysts can use the TFR system to generate more detailed, useful information as compared to using the HFDFC system alone.

## 4. Discussion

Food safety is an important global issue, and includes such things as metal exposure risk assessments. Thus, ensuring food safety of the food supply chain is a continuous challenge that requires attention [26,27]. The U.S., Taiwan, and Europe are continuing to innovate research systems such as food classification and description systems intended to improve data accuracy in data used for food safety risk assessments. In Taiwan, the food classification and description system, called the HFDFC system, has six risk-assessment-related facets, including food sources, processed products, cooking methods, manufacturers (brand), food additives, and specialty foods that are applicable to food assessment [5]. Existing food classification and description systems such as Foodex2 and the HFDFC fail to meet the needs of analysts performing risk assessments of composite foods. Given the important role of recipes in the food culture [15,16], we add to the existing food classification and description systems by proposing a supplementary food description FACET that integrates recipes into the system, called the Taiwan Food Recipe (TFR) system. Using the TFR system, analysts can reduce the estimation bias in their risk assessments. Compared with the application of a food description system on composite foods in the U.S., Taiwan, and Europe, the TFR system provides a proportion of composite food ingredients that makes it possible to calculate more detailed data. In this study, fried rice, sushi, and rice balls were considered representative of the most common high-consumption foods among Taiwan’s rice-based composite foods. The *HIs* for rice cadmium exposure risk assessment were reasonably adjusted based on the proportion of rice in the rice composite foods. However, when the TFR system was initially established, there were difficulties that had to be surmounted for the consumption data of composite foods, such as recipe quantities and data sources. The accuracy of food percentages from recipes was positively related to recipe quantities.

In the opinion of the authors, in the TFR system, the consumption data can be appropriately used on food exposure risk assessments. In this study, we further demonstrate the usefulness of the TFR system in a cadmium risk assessment risk assessment for fried rice products, sushi products, and rice ball products.

## 5. Conclusions

The construction of a standardized recipe-based food description FACET in the TFR system demonstrates more precise Cd exposure risk assessments. Further research is recommended. It is hoped that the TFR system contributes to global food classification and description systems by providing an appropriate, standardized, and generalized framework for exposure assessments.

## Figures and Tables

**Table 1:** Formulation of TFR codes for fried rice, sushi, and rice ball recipes.

HFDFC System				TFR System			
Level 1	Level 2	N of Foods	HFDFC Code	Main Ingredients in Recipe	TFR Sub-Description	TFR Code	TFR Combined Code with HFDFC
Rice composite foods [*O01*]	Fried rice [*0301*]	6	*O010301*	Rice	[whole grains]	*F07.1*	*O010301# F07.1$ F07.4$ F07.5$ F07.6$ F07.8$ F07.14*
				Anchovy larvae, Salmon	[seafood]	*F07.4*	
				Egg	[egg]	*F07.5*	
				Pork; Beef	[meat]	*F07.6*	
				Sweet pepper; Cabbage	[vegetable]	*F07.8*	
				Salt soy sauce	[seasoning]	*F07.14*	
Rice composite foods [*O01*]	Sushi (Nigiri-Sushi; Maki-Sushi) [*0302*]	31	*O010302*	Rice	[whole grains]	*F07.1*	*O010302# F07.1$ F07.4$ F07.6$ F07.8$ F07.9$ F07.14*
				Swordfish; Salmon	[seafood]	*F07.4*	
				Beef	[meat]	*F07.6*	
				Cucumber	[vegetable]	*F07.8*	
				Avocado	[fruit]	*F07.9*	
				Soy sauce; Mustard	[seasoning]	*F07.14*	
Rice composite foods [*O01*]	Rice ball [*0303*]	79	*O010303*	Rice	[whole grains]	*F07.1*	*O010303# F07.1$ F07.4$ F07.6$ F07.8$ F07.14*
				Tuna	[seafood]	*F07.4*	
				Pork	[meat]	*F07.6*	
				Tomato; Lettuce	[vegetable]	*F07.8*	
				Salt	[seasoning]	*F07.14*	
	Total	106					

**Table 2:** Formulation of TFR codes for fried rice, sushi, and rice ball recipes.

Food	Adjusted Weight ^2^									%		
	Whole Grains			Other Sub-Descriptions (g) ^1^			Total (g)			Rice	Other Sub-Descriptions	Total
	**Rice (g)**											
	Mean ± SD ^3^	Max	90%CI	Mean ±SD	Max	90% CI	Mean ±SD	Max	90% CI			
Fried rice	176.53 ± 62.41	694.10	258.13	92.35 ± 54.22	676.29	160.34	267.46 ± 34.54	434.92	312.92	65.6%	34.4%	100%
Sushi	77.23 ± 25.17	216.67	110.02	100.13 ± 29.75	291.05	138.95	176.72 ± 55.35	540.78	248.50	43.5%	56.5%	100%
Rice ball	461.07 ± 238.07	2309.01	764.92	790.04 ± 433.96	3879.03	1340.42	1229.17 ± 652.55	6781.25	2056.76	36.9%	63.1%	100%

Note. ^1^ Other TFR sub-descriptions include [Seafood], [Egg], [Meat], [Vegetables], [Fruit], and [Seasonings]. ^2^ Monte Carlo simulation for 10,000 iterations. ^3^ SD refers to standard deviation; CI refers to confidence interval.

**Table 3:** Consumption rates and cadmium Hazard Indexes for the composite products.

**Panel A: Consumption Rates**																	
Food list	**HFDFC System**			**TFR System**													
	(1)			(2)	(3)	(4) = (1) × (2)			(5) = (1) × (3)								
	*CR* for the composite product (g/day)			Rice (%)	Other Sub-descriptions (%)	*CR* for the rice ingredient in the composite product (g/day)			*CR* for the other ingredient in the composite product (g/day)			ADD for the rice in the composite product (mg/kg/day)			ADD for the other ingredients in the composite product (mg/kg/day)		
	Male	Female	Mean			Male	Female	Mean	Male	Female	Mean	Male	Female	Mean	Male	Female	Mean
Fried rice	158.48	93.63	127.5	65.60%	34.40%	103.96	61.42	83.64	54.52	32.21	43.86	0.000060	0.000043	0.000053	0.000031	0.000022	0.000028
Sushi	204.49	124.64	177.87	43.50%	56.50%	88.95	54.22	77.37	115.54	70.42	100.50	0.000051	0.000038	0.000049	0.000067	0.000049	0.000063
Rice ball	181.86	195.81	188.06	36.90%	63.10%	67.11	72.25	69.39	114.75	123.56	118.67	0.000039	0.000050	0.000044	0.000066	0.000086	0.000075
**Panel B: Cadmium Hazard Indexes**																	
	**HFDFC System**			**TFR System**													
	*HI* for the composite product			*HI* for the rice ingredient in the composite product								*HI* for the other ingredients in the composite product					
Food list	Mean	Male	Female	Mean		Male				Female		Mean		Male		Female	
Fried rice	0.09	0.09	0.07	0.06		0.06				0.05		0.03		0.03		0.02	
Sushi	0.10	0.12	0.09	0.04		0.05				0.04		0.06		0.07		0.05	
Rice ball	0.13	0.10	0.14	0.05		0.04				0.05		0.08		0.06		0.09	

Note. RfD (mg/kg/day) = 0.001, US EPA, ORD number CASRN 7440-43-9. C = Concentration in food =0.04 mg/kg. *CR* is Consumption rate = Actual consumption intake x estimated percentage of the average cooked weight of each food ingredient from all recipes in the composite food. *HI* = Hazard Index. The mean value of body weight for subjects between the ages of 19–65 is 63.3 kg. Male and female body weight for subjects between the ages of 19–65 is 69.33 kg and 57.27 kg, respectively. Monte Carlo simulation for 10,000 iterations.
